# Supplementary material for: Interstitial Deletion of 3q21 in a Kuwaiti Child with Multiple Congenital Anomalies—Expanding the Phenotype
Source: Genes (Basel). 2023 Jun 5;14(6):1225. doi: 10.3390/genes14061225 (PMC10298411; doi:10.3390/genes14061225)
Supplement: Supplementary file 1 [file genes-14-01225-s001.zip › genes-2311187-supplementary.pdf]

Gene	OMIM phenotype number	Location	Inheritance	Phenotype
MCM2	616968	3q21.3	AD	Deafness
ADCY5	606703	3q21.1	AD	Dyskinesia, facial myokymia
CASR	239200	3q13.3-q21.1	AD	Hyperparathyroidism, neonatal
	145980		AD	Hypocalciuric hypercalcemia, type I
	601199		AD	Hypocalcemia with Bartter syndrome
	612899		AD	Epilepsy idiopathic generalized
ZNF148	617260	3q21.2	AD	Global developmental delay, absent or hypoplastic corpus callosum, and dysmorphic features
SEC61A1	617056	3q21.3	AD	Tubulointerstitial kidney disease
RAB7A	600882	3q21.3	AD	Charcot-Marie-Tooth disease, type 2B
MYLK	613780	3q21.1	AD	Aortic aneurysm, familial thoracic 7
GATA-2	614038	3q21.3	AD	Emberger syndrome
	614172		AD	Immunodeficiency 21
	601626		AD	Susceptibility to acute myeloid leukemia
	614286			Susceptibility to myelodysplastic syndrome

Supplementary Table 1. OMIM genes associated with disease in the long arm of chromosome 3 with autosomal dominant (AD) inheritance.
